# From Design to Manufacture of a Carbon Fiber Monocoque for a Three-Wheeler Vehicle Prototype

**DOI:** 10.3390/ma12030332

**Published:** 2019-01-22

**Authors:** Alessandro Messana, Lorenzo Sisca, Alessandro Ferraris, Andrea Giancarlo Airale, Henrique de Carvalho Pinheiro, Pietro Sanfilippo, Massimiliana Carello

**Affiliations:** Department of Mechanical and Aerospace Engineering, Politecnico di Torino, 10129 Turin, Italy; alessandro.messana@polito.it (A.M.); lorenzo.sisca@polito.it (L.S.); alessandro.ferraris@polito.it (A.F.); andrea.airale@polito.it (A.G.A.); henrique.decarvalho@polito.it (H.d.C.P.); pietro.sanfilippo1990@gmail.com (P.S.)

**Keywords:** carbon fiber, structural analysis, monocoque structure, lightweight design, low consumption vehicle, three-wheeler vehicle

## Abstract

This paper describes the design process of the monocoque for IDRAkronos, a three-wheeler hydrogen prototype focused on fuel efficiency, made to compete at the Shell Eco-Marathon event. The vehicle takes advantage of the lightweight and high mechanical performance of carbon fiber to achieve minimal mass and optimized fuel consumption. Based on previous experiences and background knowledge, the authors describe their work toward a design that integrates aerodynamic performance, style, structural resistance and stiffness. A portrayal of the objectives, load cases, simulations and production process—that lead to a final vehicle winner of the Design Award and 1st place general at the 2016 competition—is presented and discussed.

## 1. Introduction

The importance of lightweight design and high mechanical performance in contemporary automotive development is undisputed. Certainly, one of the main related goals is fuel efficiency.

In order to achieve a good compromise between mass, mechanical properties, aerodynamic performance and style, the use of carbon fiber stands out. Its application allows creative shapes to couple with elevated engineering performance, permitting high design freedom.

The main issues of carbon fiber technology are the cost of production and the recyclability; therefore, its major applications still lie in the racing field and niche markets. 

This paper presents the design and manufacturing of a three-wheeler prototype for the Shell Eco-marathon competition [1], which awards the vehicle that obtains the lowest fuel consumption measured in terms of km, with a liter equivalent fuel (calculated by Shell, using chemical formulations).

Starting from experience in other vehicles designs [2,3,4] at the Politecnico di Torino and also by other teams [5], the new vehicle—IDRAkronos—has been developed, using a carbon fiber monocoque to reach minimal mass, while maintaining structural resistance. The prototype has three wheels; two front steering wheels (covered by the body to reduce aerodynamic drag) and one rear wheel powered by a brushed electric motor, supplied by a hydrogen fuel cell. The upper limit of 40 kg has been respected, including by all the sub-systems; steering, breaks, wheels, cockpit, electric wiring, controls, fuel cell, the electric motor, and transmission.

During the thorough technical inspection that takes place before the competition [1], one of the most challenging tests for the vehicle body was the application of a 70 kg load on the highest point of the vehicle (roll bar). The structure of the vehicle should not deform permanently, nor show ruptures during the test. To withstand this load, a good technological solution is the carbon fiber composite, which has excellent performance in terms of low density and mechanical properties [6,7], allowing us to attain minimum mass and high resistance, but also to make an aerodynamically optimized shape.

Apart from the technical regulation constrains and some geometrical fixed features (for instance, the vehicle track) the design followed was conducted in order of CAD (Computer Aided Design), FEM (Finite Element Method) analysis, CFD (Computational Fluid Dynamics) analysis and some iteration between structural resistance and aerodynamic performance. Once the final shape was achieved, a definitive FEM model was developed, concurrently with the ply book for the manufacturing process.

A typical manufacturing technology used to produce carbon fiber components for automotive applications is the use of pre-pregs, which are fabric composite materials already littered with resin. The pre-pregs are shaped manually in molds and then polymerized in an autoclave, using a vacuum bag around the mold. Unidirectional and multi directional carbon fiber fabrics were used, with a specific ply lay-up to balance the stress due to pressure and temperature variation, which occurs during the curing cycle in the autoclave. By doing so, it is possible to obtain components with elevated uniformity and performance.

## 2. Materials and Methods

### 2.1. Geometry Design

**Figure 1:**
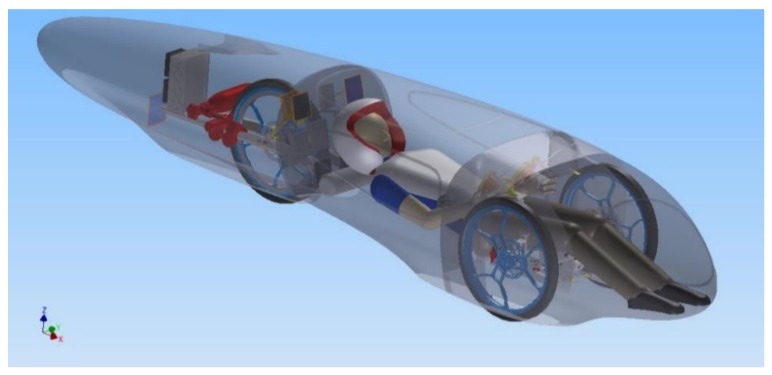
IDRAkronos’ layout.

A significant change in IDRAkronos’ layout (Figure 1)—with respect to the prototypes designed previously [2,3,4]—is the location of the rear wheel behind the firewall, and the fuel cell with the hydrogen tank at the end of the car. This solution grants not only a considerably stiffer rear assembly and a shorter wheelbase, but also higher design freedom for the external shape of the vehicle’s tail. Moreover, this position enhances the ventilation of the fuel cell, increasing the powertrain’s efficiency.

The position of the driver was set such that the steering bar passed below the knees, while the wheels were leveled with the hip. The choice of the vehicle’s height was the result of a compromise, where on the one hand, a low height would reduce the frontal area (and therefore the aerodynamic drag) and avoid rollover, but a more elevated floor would prevent the downforce caused by ground effects, thus reducing the rolling resistance.

The engine was directly mounted on the chassis in order to minimize relative motion between the pinion and the gear mounted on the wheel. This way, a better coupling efficiency was achieved during cornering, where the components typically tend to move apart.

Moreover, IDRAkronos had three particularly well-developed characteristics:

1. Covered front wheels that reduced turbulence generated by the spinning motion. In this case, the wheel arch volume had to include the envelope of the steering wheels, increasing the frontal area of the car, which was compensated for, by a significant reduction in the drag coefficient.
2. Reduced wheelbase of the vehicle helps decreased the frontal area of the car and also the volume of the wheel arches, improving the aerodynamic resistance (a lower wheelbase means a lower steering angle is needed to run the same curve). Another advantage of the reduced wheelbase is that the length of the structural part of the car was shortened, allowing a reduction of the bending stresses on the monocoque, and thus helping to achieve a lighter vehicle.
3. Increased length of the body allowed us to close the tail with a smaller angle, delaying the transition from laminar to turbulent flow as far back as possible, reducing the dimensions of the wake, and therefore the drag coefficient.

**Figure 2:**
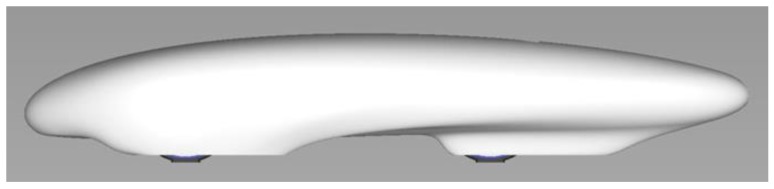
IDRAkronos’ external shape.

The software used to design the surfaces is Alias (2015 Version) by Autodesk, San Rafael, CA, USA. The design of IDRAkronos (Figure 2) was influenced by profile 4415 of NACA (National Advisory Committee for Aeronautics) airfoil, with dimensions according to their characteristics and the compatibility with the proportions of the prototype. The NACA duct was designed to work with a rear opening which allows the air inside the rear compartment to flow outside, avoiding the spoon effect. The refrigeration of the fuel cell was one of the main objectives; in particular, the temperature profile was estimated on its duty cycle during the race, at different times of the day. The temperature gap between the two solutions justified the presence of the NACA duct in order to improve the powertrain’s efficiency.

**Figure 3:**
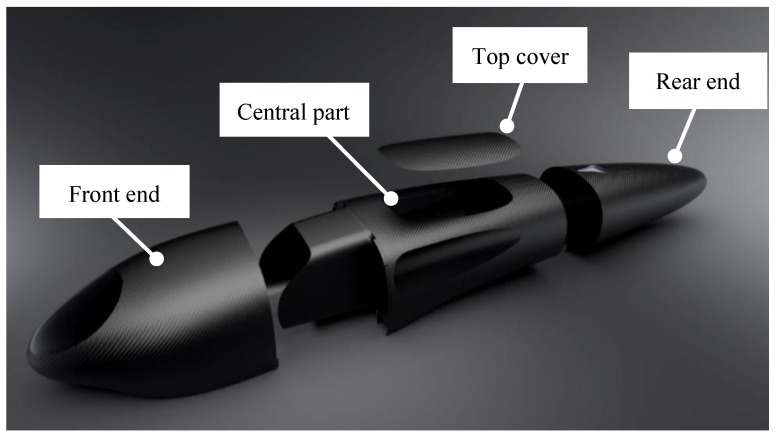
Subdivisions of the IDRAkronos’ body.

The body of IDRAkronos is subdivided into the following parts (Figure 3):

- The front end
- The central part
- The rear end
- The top cover

The front end, rear end, and the cover did not add a relevant contribution to the stiffness of the vehicle, and the central part had the purpose of withstanding all static and dynamic loads that acted on the vehicle.

With IDRAkronos, the objective was to have a closed cross section in order to significantly improve the moment of inertia of the monocoque, improving the stiffness due to the geometry factor, and therefore allowing us to use less material to achieve the same displacements.

### 2.2. Materials

The monocoque of IDRAkronos is made of 0.25 mm thick sheets of 2 × 2 twill T300 carbon fiber/epoxy pre-preg, layered in a sandwich structure within the Nomex honeycomb. The mechanical properties of these materials are reported in Table 1. Carbon fiber based composites show a high strength-to-volume ratio, which make them ideal for applications that need both lightness and high strength. Preferred orientations used for pre-preg sheets were 0°, 45°, −45°, and 90°, while avoiding 30° and 60° for simplicity during the manufacturing process.

The sandwich structure is frequently used in the motorsport and aerospace industry due to its ability to combine the best properties of both materials. Nomex honeycomb is a core material made up of aramid polyamide fibers, which have low specific mass and high torsional resistance, impact toughness and vibration damping. The possibility of using a core material with very low density allowed us to employ a higher thickness core (ranging from 6 mm to 13 mm in this application, as shown in Figure 10) and, importantly, to increase the moment of inertia of the section without adding too much mass.

### 2.3. FEM Model

**Figure 4:**
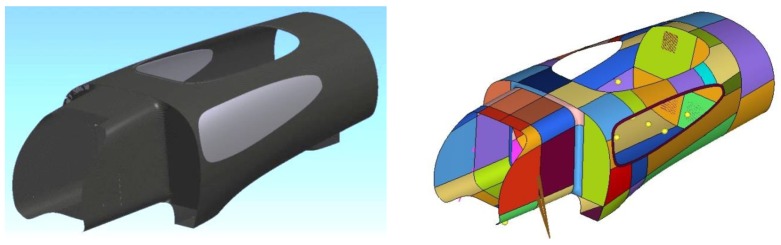
Central part CAD design (**left**) and subdivision to enhance mesh quality (**right**).

**Figure 5:**
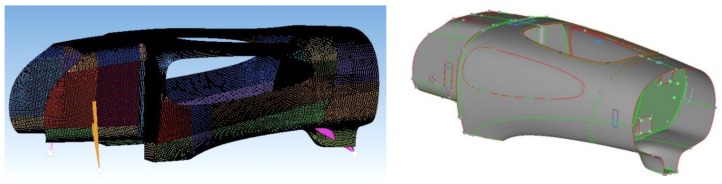
FEM shell elements mesh definition (**left**) and pre-processing geometry cleanup (**right**).

Linear static analyses of IDRAkronos were made using the Hypermesh and Hyperview software (v.14) and using the Optistruct solver (both provided by Altair, Troy, MI, USA). Figure 4 shows the CAD design of the body and its smaller subdivisions, with different sizes and curvature used to better analyze and control the FEM mesh quality. In the pre-processing interface, the geometry clean-up and meshing process was carefully carried out, as depicted in Figure 5.

Two-dimensional elements have been used for the mesh, since one dimension is negligible with respect to the others for the panels, under the hypothesis that the distribution of stresses along the axis orthogonal to the element plane is irrelevant. Although stiffer than quadrilateral elements, triangular elements were necessary to follow the fairly complex geometry. The quality of an element was determined by minimum length, skew angle, Jacobian, aspect ratio, and warpage. In fact, reducing the size of the elements increased their number, so results were more accurate with a longer computational time.

MAT 8 was used in Hypermesh to define linear temperature-independent orthotropic materials for two-dimensional elements. The PCOMPP card was used in combination with the STACK and PLY cards to create composite properties through the Ply-based composite definition.

Composite materials are highly anisotropic and behave differently based on their orientation. The 0° direction was oriented along the x-axis of the vehicle.

The track on which the race was held was analyzed and, due to the speed profile expected during the race, a target on the limit for rollover conditions was set, assuming infinite adherence from the tires. From the Shell Eco-marathon regulations [1], the vehicle was required to be able to run a 10 m radius curve at a speed of up to 27 km/h. Multibody dynamic simulations made with Adams View confirmed the above requirements, outputting the forces acting on the wheels right before rollover (Table 2).

In regards to the other loads, the mass of the head was applied in a rectangular shape onto the firewall, since the head was placed above the fire extinguisher-holding structure. The loads on the back and hips were applied on an area with a width roughly equal to the shoulder width of the driver, and the loads caused by the driver’s feet were applied on the front end of the vehicle. Constraints, similar to those on the load step were placed, which assured that the driver was inside the vehicle.

Furthermore, the firewall had the function of a roll bar, which had to be capable of withstanding a static load of 700 N, as the regulations stated.

## 3. Results

### 3.1. FEM Results

The design phase was composed of three principal steps; free-size-optimization, discrete-size-optimization, and shuffle-optimization. As the monocoque of IDRApegasus (the predecessor of IDRAkronos) never reached more than 18 mm in thickness, the maximum thickness of the laminate was set to 20 mm during optimization. Total displacement response to the load steps “driver inside, stand, and exit” was considered to be 1 mm. For most parts, two plies of carbon fiber for each orientation were more than enough, and the minimum thickness for each orientation was, in fact, set to 0.5 mm, corresponding to two plies, in order to keep the laminate balanced and guarantee a certain degree of isotropy.

To improve correlation with the real application and reduce to bare minimum the amount of material used to obtain the preset target in terms of stiffness, the contribution of the side windows was computed inside the FEM model. This is an essential step of the design and optimization process, since an inaccurate description of the general behavior of the structure could lead to the exaggeration of the thickness (and therefore weight) of the body.

**Figure 6:**
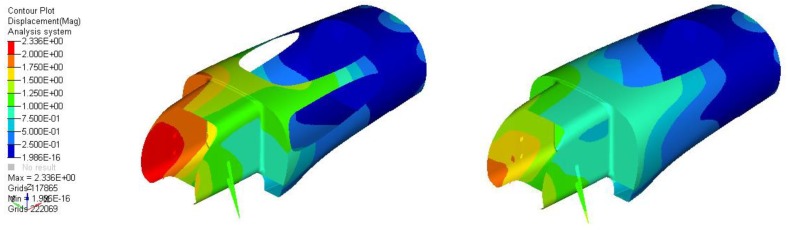
Rollover displacements without (**left**) and with (**right**) windows.

**Figure 7:**
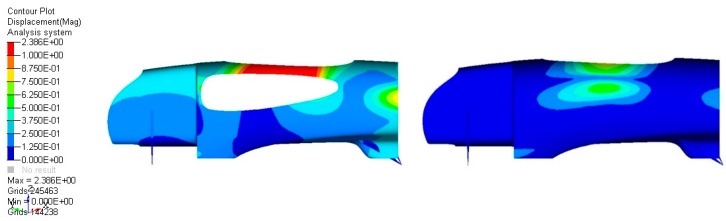
Exit displacements without (**left**) and with (**right**) windows.

For optical reasons, 3 mm thick scratch-resistant antireflection-certified panels made by Lexan were used, resulting in a 1.5 kg mass addition to the vehicle. The lateral windows’ contribution to the bending stiffness of the monocoque was very important, as depicted by Figure 6, which shows the effect on the roll-over conditions, and Figure 7, which shows the effect on the displacement due to the driver’s hold during the exit from the vehicle. It is clear, after these results, that the inclusion of the glass contributed majorly to the structural performance, allowing a proper setup for the optimization and demonstrating that these elements should be carefully designed and installed.

The same approach has been used for the design of the front end and the tail of the vehicle, with lower loads.

**Figure 8:**
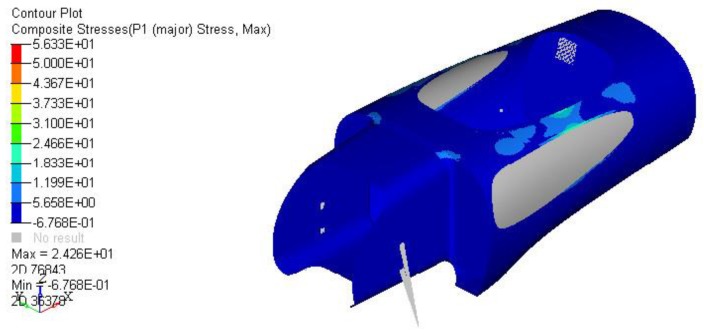
Stress distribution during the exit maneuver.

Given strict requirements from the displacement point of view, stresses remained very low, of the order of few MPa, as shown in Figure 8. Nonetheless, for the sake of completeness, a ply failure check was computed using Tsai Wu theory for composite materials. As expected, ply failure never occurred in any load step, even in the most severe tests of the monocoque.

**Figure 9:**
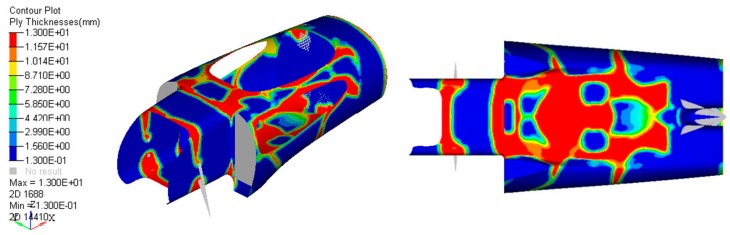
Free-size-optimization of honeycomb thickness: Top (**left**) and bottom (**right**) view.

**Figure 10:**
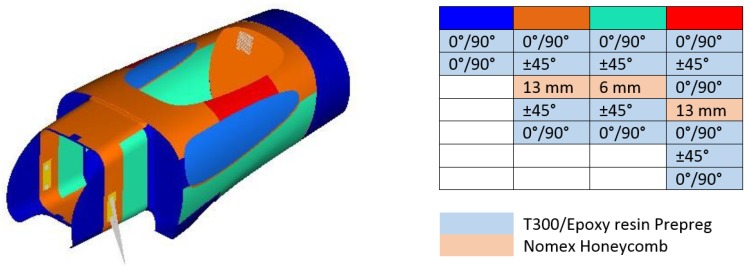
Final laminate (**left**) and stacking sequence (**right**).

Many analyses, such as the free-size-optimization (Figure 9), were carried out in order to define the best shapes and thicknesses of the honeycomb inside the body. This was extremely important in order to achieve the goal of minimum mass (and ultimately lower consumption) for the vehicle, while maintaining mechanical performance. Bearing in mind that the limits on the thicknesses available were only 13 mm, 6 mm, and 3 mm, and the shapes that could be obtained due to different curvatures of the monocoque, the final lamination ply book is reported in Figure 10.

### 3.2. Production Process

The final properties of the components in composite materials, other than the properties of the carbon fiber and matrix, depend strongly on the production process. For this reason, it was not enough to choose the right type of materials, it was also of great importance to evaluate the appropriate production process in order to guarantee good quality of the final product. Inter-laminar cohesion, for example, was strictly dependent on the absence of air bubbles absorbed during the manufacturing of the laminate.

The most suitable process to realize the components of the prototype was the use of the autoclave vacuum bag, which required a phase of composite lamination in a mold and a phase of thermal consolidation both in vacuum and under pressure. 

**Figure 11:**
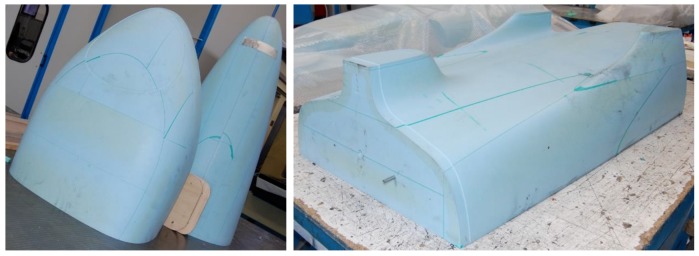
Male resin molds; front and rear end (**left**), and central part (**right**).

**Figure 12:**
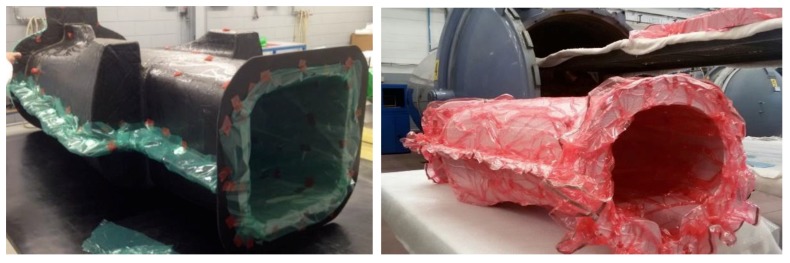
First lamination: Release film (**left**) and vacuum bag (**right**) on the female central mold.

Blocks of epoxy resin (Figure 11) RAKU-TOOL WB0691 (Rampf Company, Gräfenberg, BY, Germany) were used to reduce the costs of metal. Resin male molds were used to realize carbon fiber female molds, which carried out the first carbon fiber lamination. Figure 12, on the left, shows the closure of the female molds in the release film, and Figure 12, on the right, shows the closure of the breather film and vacuum bag before the autoclave cycle. The autoclave cycle used lasted 3 h, at 130 °C and 5.5 bar.

**Figure 13:**
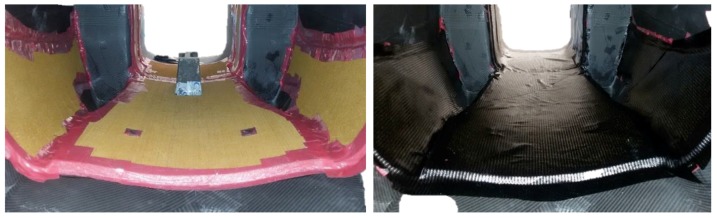
Second lamination in the central part: Nomex honeycomb (**left**) and composite layers (**right**).

For the second lamination, honeycomb sheets were glued in the central part of the body, only where designed by FEM (Figure 13, left). The composite layers were laminated to realize the internal shell (Figure 13, right). In order to avoid the collapse of Nomex, the second curing cycle was done at a maximum of 1.5 bar.

**Figure 14:**
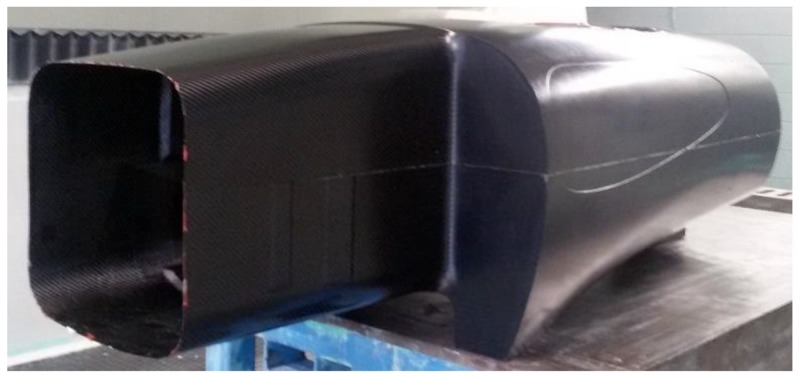
Final central part.

After the body was released from the mold (Figure 14), some trimming was done on the tunnel, the windows, and the wheel mounting zone. To obtain a perfectly vertical laminate needed to mount the wheel, six of 22 plies of carbon fibers were sacrificed in the milling machine. Before starting the trim, the body was properly positioned and the symmetry plane was traced. This was crucial, so as to have the faces, on which the wheel brackets had to be mounted, perfectly parallel to the vehicle’s z–x plane, with an offset of ±210 mm along the y- axis, and therefore to minimize the error on the final wheel alignment. Finally, all the holes were drilled, the metal inserts screwed in, and the hooks affixed on the monocoque.

The manufacture of the firewall was relatively simple, with only one curing cycle of 12 mm of honeycomb sheets between carbon fiber plies. To properly glue the firewall on the monocoque, a guide was built with four centering pins placed on the main body to guarantee the perfect placement of the firewall at exactly 15° from the vertical plane. An error at this stage could have jeopardized the position of the rear wheel, both in relative misalignment and in absolute positioning, leading to a permanent, unwanted pitch or roll angle of the whole vehicle.

The front end was laminated with two plies, while four plies were placed in the frame in contact with the main body, to increase the stiffness and the handling. The rear end was laminated with two plies, and after curing, the only operation made on the rear end was the cutting of a hole used to glue the NACA duct, which was 3D printed previously. 

## 4. Discussion

**Figure 15:**
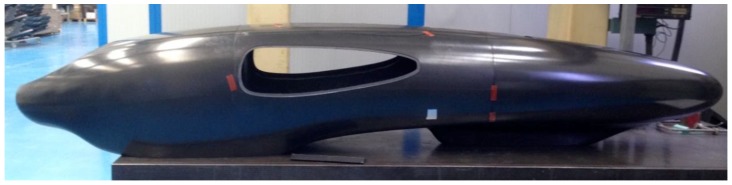
Full vehicle body assembled.

Mass reduction is a key factor in the design of a prototype vehicle aimed at a low consumption competition, such as the Shell Eco-marathon. In this paper, the structural design of IDRAkronos prototype’s body, from the concept phase (Figure 3) to the manufacturing process (Figure 15) was illustrated. Trying to minimize the mass of the vehicle, the authors highlighted the main steps of the CAD design that creates the general shape, the aerodynamic requirements, and finally the FEM simulations and optimization process. In this way, the ply book of the composite was defined before fabricating the body using the pre-preg, hand lay-up, and autoclave production processes.

The design of the carbon fiber monocoque delivered great results in terms of mass and stiffness. The technical solutions adopted were proved to be successful, and each detail was important in achieving the desired mechanical performance of the structure, and therefore the general objectives for the whole vehicle. The main body had a mass of 7.5 kg, a tail mass of 1.2 kg, while the front-end mass was 2.5 kg. This lightweight design contributed to obtaining a total vehicle mass of just 39 kg.

The design methodology and insights provided by this paper may contribute to the development of similar low consumption vehicles, creating a guideline for the application of carbon fiber composite materials in monocoque structures [8,9,10], or any other component with similar complexity and variety of constrains and goals.

## Figures and Tables

**Table 1:** Mechanical properties of carbon fiber laminate and Nomex honeycomb.

Property	T300/Epoxy Composite	Nomex Honeycomb
E—Young’s Modulus (GPa)	57	0.9
G—Shear Modulus (GPa)	3	0.3
σ_t_—Tensile Strength (MPa)	570	-
σ_c_—Compressive Strength (MPa)	530	80
ν—Poisson’s Ratio	0.05	0.4
ρ—Specific Mass (kg/dm^3^)	1.4	0.06

**Table 2:** Rollover forces.

Tire	F_z_ [N]	F_y_ [N]
External front	650	350
Internal front	0	0
Rear	350	150
